# A Binding-Site Barrier Affects Imaging Efficiency of High Affinity Amyloid-Reactive Peptide Radiotracers In Vivo

**DOI:** 10.1371/journal.pone.0066181

**Published:** 2013-06-04

**Authors:** Jonathan S. Wall, Angela Williams, Tina Richey, Alan Stuckey, Ying Huang, Craig Wooliver, Sallie Macy, Eric Heidel, Neil Gupta, Angela Lee, Brianna Rader, Emily B. Martin, Stephen J. Kennel

**Affiliations:** 1 Department of Medicine, University of Tennessee Graduate School of Medicine, Knoxville, Tennessee, United States of America; 2 Department of Radiology, University of Tennessee Graduate School of Medicine, Knoxville, Tennessee, United States of America; 3 Department of Surgery, University of Tennessee Graduate School of Medicine, Knoxville, Tennessee, United States of America; Nathan Kline Institute and New York University School of Medicine, United States of America

## Abstract

Amyloid is a complex pathology associated with a growing number of diseases including Alzheimer’s disease, type 2 diabetes, rheumatoid arthritis, and myeloma. The distribution and extent of amyloid deposition in body organs establishes the prognosis and can define treatment options; therefore, determining the amyloid load by using non-invasive molecular imaging is clinically important. We have identified a heparin-binding peptide designated p5 that, when radioiodinated, was capable of selectively imaging systemic visceral AA amyloidosis in a murine model of the disease. The p5 peptide was posited to bind effectively to amyloid deposits, relative to similarly charged polybasic heparin-reactive peptides, because it adopted a polar α helix secondary structure. We have now synthesized a variant, p5R, in which the 8 lysine amino acids of p5 have been replaced with arginine residues predisposing the peptide toward the α helical conformation in an effort to enhance the reactivity of the peptide with the amyloid substrate. The p5R peptide had higher affinity for amyloid and visualized AA amyloid in mice by using SPECT/CT imaging; however, the microdistribution, as evidenced in micro-autoradiographs, was dramatically altered relative to the p5 peptide due to its increased affinity and a resultant “binding site barrier” effect. These data suggest that radioiodinated peptide p5R may be optimal for the *in vivo* detection of discreet, perivascular amyloid, as found in the brain and pancreatic vasculature, by using molecular imaging techniques; however, peptide p5, due to its increased penetration, may yield more quantitative imaging of expansive tissue amyloid deposits.

## Introduction

Amyloid is a proteinaceous aggregate associated with a growing number of diseases and with aging [1]–[3]. Some systemic amyloidoses (e.g., light chain [AL], serum amyloid protein A [AA] and transthyretin [ATTR]) are rare, whereas other visceral amyloid-related diseases (e.g., type 2 diabetes and dialysis arthropathy) are far more common. The former are often incurable disorders that are likely under-diagnosed due to the heterogeneity in presentation. The latter diseases are more common, and the importance of amyloid deposits in disease etiology is still being established as it was in Alzheimer’s disease a decade ago [4].

Currently, there are no FDA-approved agents available in the USA for quantitative whole body imaging of visceral amyloid *in vivo*. Recent data suggest that small molecule tracers, such as Pittsburgh compound B (PIB) which is used for Aβ amyloid imaging in patients with Alzheimer’s disease, may prove useful for the routine clinical detection of cardiac amyloidosis [5]. However, there is no evidence that either this or the recently FDA-approved Aβ tracer Amyvid™ will facilitate detection of amyloid deposits in other visceral organs. Numerous amyloid-binding radiotracers have been described, e.g. ^99m^Tc-aprotinin [6]–[8], ^99m^Tc-DPD [9], [10], ^131^I-β_2_M [11], [12], and ^123^I-SAP [13]–[15]. However, only ^123^I-SAP whole body, planar scintigraphic imaging of amyloid, developed approximately 2 decades ago at the Royal Free Hospital in London, is in routine clinical use [14], [15]. This method is not available in the US because the SAP is of human origin and, due to its structural sensitivity, cannot tolerate the stringent viral inactivation steps required by the FDA. In addition, cardiac and pancreatic amyloid is, at best, poorly detected using SAP imaging.

Imaging visceral amyloidosis in patients with diseases such as type 2 diabetes, rheumatoid arthritis and myeloma may provide the physician with an additional tool to assist in the diagnosis of these diseases. Images can provide information on the whole body disease distribution and burden without the need for multiple invasive biopsy procedures. Thus, molecular imaging could help inform treatment strategies and prognostication (e.g. in patients with type 2 diabetes, the IAPP amyloid load may act as a surrogate for β-cell loss in the pancreatic islets - [16]). Perhaps most importantly, the detection of amyloid by molecular imaging can be used to stratify patients for new treatments and to monitor the response to therapies quantitatively, avoiding unnecessarily expensive, toxic or ineffective treatment.

To this end, we have characterized a peptide, designated p5, that could be used to specifically image the distribution of AA amyloid in a mouse model of the disease by using dual single photon emission and x-ray computed tomographic (SPECT/CT) imaging and microautoradiography [17]. Dual energy SPECT imaging showed that p5 was comparable to SAP for imaging AA murine amyloid in liver and spleen. Furthermore, pancreatic and intestinal amyloid deposits were imaged with p5 but not with SAP [18]. Importantly, the amount of uptake of radiolabeled p5 in hepatic and splenic AA amyloid deposits was shown to correlate with the amyloid load in those organs as evidenced by Congo red staining and quantification [19].

The *in vivo* amyloid imaging efficacy of peptide p5 was postulated to be due to its ability to adopt a polar α helix that resulted in the presentation of a linear array of basic lysine side chains with a regular periodicity [17]. To test this hypothesis and to identify a potentially better imaging peptide, we have generated an arginine-substituted variant of p5, p5R, that was postulated to have a higher propensity for α-helical formation and, therefore, enhanced amyloid binding capabilities *in vivo* along with improved imaging characteristics. Herein, we describe the characterization of peptide p5R which exhibited increased affinity for both immobilized heparin and AA amyloid-laden tissue extracts. However, when administered to mice with severe AA amyloid, the microdistribution of the p5R peptide within the splenic amyloid was dramatically altered due to a binding site barrier effect.

## Materials and Methods

### Ethics Statement

These studies were carried out in strict accordance with a protocol approved by the University of Tennessee Institutional Animal Care and Use Committee. All procedures were approved by the IACUC and were performed in accordance with the guidelines provided by OLAW and the Guide for the Care and Use of Laboratory Animals.

### Preparation of peptides p5, p5R and p5R(1–24)

Peptides were purchased from Keck Laboratories and further purified by high performance liquid chromatography (Agilent, 1100 series) by elution from a reverse-phase C3 matrix in a linear gradient of 0 to 50% acetonitrile in water with 0.05% trifluoroacetic acid (flow rate of 1 mL/min). One mL-fractions were collected, pooled and the mass verified by mass spectrometry using a single quadrupole MS (Applied Biosystems).

### Murine model of AA amyloidosis

AA amyloidosis was induced in H2-Ld-huIL-6 Tg Balb/c transgenic mice that constitutively express the human interleukin-6 transgene [20], [21] by iv administration of 10 µg of isolated amyloid enhancing factor (AEF – a suspension of AA amyloid fibril extract, [22]) in 100 µL of sterile phosphate-buffered saline (PBS). Mice used in these studies were 4–6 wk post induction. These studies were carried out in strict accordance with a protocol approved by the University of Tennessee Institutional Animal Care and Use Committee. All procedures were approved by the IACUC and were performed in accordance with the guidelines provided by OLAW and the Guide for the Care and Use of Laboratory Animals.

### Circular dichroism spectroscopy

CD spectra of peptides p5 and p5R (0.05 mg/mL in PBS) were acquired using a DSM 1000 CD instrument (Olis Inc., Bogart, Georgia) with a peptide sample volume of 2.9 mL and a 1 cm cuvette path length. Data were collected in triplicate over the 190 nm – 250 nm wavelength range with 1 nm increments. Coil to helix transitions were induced by preparing peptides at 0.05 mg/mL in PBS with 2,2,2 trifluoroethanol up to 40% by volume. Spectra were corrected for background by subtraction of a PBS-only CD spectrum. Mean residue ellipticity [θ] was calculated according to:

[θ] = θ*(MW/*no. AA*)/(10*conc.*l)

where: *θ* is elipticity (millidegrees); MW is the molecular weight of the peptide; *no. AA*, is the number of amino acid residues; *conc*. is the peptide concentration (mg/mL), and; *l*, is the cuvette pathlength (cm)

### Solid phase heparin binding studies

Assessment of solid-phase heparin binding was performed using a 20 mL bed-volume Heparin FF16/10 column (GE Healthcare #28-9365-49) with a BioRad Biologic duoflow FPLC system. An aliquot of 0.5 mg of peptide (1 mg/mL) was injected over 30 sec onto the heparin column and eluted using a gradient from 0 – 2 M NaCl over 10 minutes with a flow rate of 2 mL/min. The absorbance at 280 nm and the conductivity (mS/cm) were measured using in-line detectors.

### Radiolabeling of peptides

Peptides (100 µg) were radioiodinated with ∼ 2 mCi of iodine-125 or -123 (^125^I, Perkin Elmer, Waltham, MA; ^123^I, MDS Nordion, Ottawa, ON, Canada), using 20 µg chloramine T. After quenching the reaction with 20 µg sodium metabisulfite, the radiolabeled peptides were diluted into 0.1% sterile gelatin in PBS and purified by gel filtration on a 5 mL PD10 column equilibrated in gelatin/PBS. Peak fractions of radiotracer were pooled and the product’s radiochemical purity was established by SDS polyacrylamide gel electrophoresis (PAGE) analysed by phosphor imaging (Cyclone Storage Phosphor System, Perkin Elmer, Shelton, CT).

### Ex vivo binding to AA amyloid extract

The livers of 2 mice with reactive (AA) amyloidosis and 2 healthy (WT) mice were harvested at necropsy. Approximately 0.5 g of each AA or WT liver was removed, pooled, and a 10-fold volume of PBS added. Serine protease inhibitors leupeptin and phenylmethylsulfonyl fluoride (PMSF) were added, each at 100 µg/mL. The solution was mixed vigorously (3×10 s bursts) with a Polytron (Kinematica Inc., Bohemia, NY) and then centrifuged at 4,000 x g for 10 min. The supernatant was discarded and the pellet resuspended in a 10-fold volume of PBS with 0.05% tween-20 and 0.05% NaN_3_. The solution was mixed vigorously again by using the Polytron and the resulting suspension stored at 4°C.

FiftyµL (∼5 mg) of liver extract was centrifuged at 21,000 x g for 3 min. The supernatant was discarded and the pellet resuspended in 400 µL PBS with 0.05% tween-20 (PBST). TwentyµL of a 1∶100 dilution of radiolabeled peptide ^125^I-p5 or ^125^I-p5R (∼ 200,000 cpm) was added to the suspension. The mixture was rotated at RT for 1 h before being centrifuged at 10,000 x g for 3 min. Supernatants, containing unbound peptide, were collected in glass tubes and the pellets washed by addition of 400 µL PBST and centrifugation at 10,000 x g for 3 min. The supernatants were again removed and added to those previously collected. Pellets were resuspended in 800 µL of PBST and transferred to glass tubes. The radioactivity in each sample was measured using a Packard Cobra II gamma counter (Perkin Elmer, Waltham, MA) with a 1 min acquisition time. The percentage of radiolabeled peptide bound to the pellet was determined as follows where CPM is counts per minute:

[*Pellet CPM*/(*Pellet CPM* + *Supernatant CPM*)] * 100

To assess the relative affinity of the peptides for AA liver homogenate, the binding of ^125^I-p5 and ^125^I-p5R was performed as described above, in 0.15, 0.25, 0.5, 0.75 and 1 M NaCl (0.05% tween-20). To generate binding data for Scatchard analysis 5 ng (∼70,000 cpm) of radioiodinated peptide was added to the tissue homogenate with increasing amounts of unlabeled homologous peptide (up to 2 µg for p5 and 1 µg for p5R).

### In vivo amyloid reactivity of peptides p5, p5R and p5R(1–24)

Mice, wild type or those with AA-amyloid, were injected iv in the lateral tail vein with ∼10 µg (∼150 µCi) of radioiodinated (^123^I or ^125^I) peptide p5, p5R, p5R(1–24) or p5R mixed with 1 mg of unlabeled p5R peptide. After the appropriate uptake time, 1, 2, or 4 h (see figure legend), the mice were euthanized by an isoflurane inhalation overdose. At this time, SPECT/CT images were acquired followed by a necropsy performed for biodistribution and autoradiographic analyses.

### In vivo biodistribution measurements

Samples of muscle, liver, spleen, pancreas, kidneys, heart, stomach, small and large intestines and lung tissue were harvested post mortem from every mouse [23]. Each sample was placed into a tared, plastic vial, weighed and the ^125^I or ^123^I radioactivity measured using an automated Wizard 3 gamma counter (1480 Wallac Gamma Counter, Perkin Elmer). The biodistribution data were expressed as % injected dose/g tissue (% ID/g). When dual-energy biodistribution measurements were performed, the high energy gamma rays of ^123^I were windowed, and this value was used to correct for spill down of the ^123^I photons into the lower energy ^125^I window. Standard samples of unmixed probes were used to determine cross-over parameters and also for %ID/g calculations. In addition, samples of each tissue were fixed in 10% buffered-formalin for 24 h and embedded in paraffin for autoradiography.

### Autoradiography

Four to 6 μm-thick sections were cut from formalin-fixed, paraffin-embedded blocks onto Plus™ microscope slides (Fisher Scientific), dipped in NTB-2 emulsion (Eastman Kodak), stored in the dark and developed after a 96-h exposure. Each section was counter-stained with hematoxylin. Amyloid deposits were confirmed after staining with alkaline Congo red. Tissues were examined using a Leica DM500 light microscope fitted with cross-polarizing filters. Digital microscopic images were acquired using a cooled CCD camera (SPOT, Diagnostic Instruments, Sterling Heights, MI).

### SPECT/CT imaging of ^125^I-p5R in AA mice

Imaging was performed as described [18]. Briefly, WT or AA-amyloid mice were injected with ∼7.5 µg of ^125^I-p5R (∼ 150 µCi) in the lateral tail vein. After a 2 h uptake time, the mice were euthanized by an isoflurane inhalation overdose.

SPECT images were acquired using an Inveon trimodality imaging platform (Siemens Preclinical Solution, Knoxville, TN; [24]). Low energy (^125^I; 25 – 45 keV) gamma photons were acquired at each of 60, 16-sec projections with 90 mm of bed travel. A 1 mm-diameter 5-pinhole (Mouse Whole Body) collimator was used at 30 mm from the center of the field of view. Data were reconstructed post hoc onto an 88×88×312 matrix with isotropic 0.50 mm voxels using a 3D ordered subset expectation maximization (OSEM) algorithm (8 iterations; 6 subsets). CT data were acquired using an x-ray voltage biased to 80 kVp with a 500 µA anode current, and the data were binned at 4×4. A 225 msec exposure was used, and 360, 1-degree projections were collected. The data were reconstructed using an implementation of the Feldkamp filtered back-projection algorithm [25] onto a 512×512×1296 matrix with isotropic 0.106 mm voxels. SPECT and CT datasets were visualized using the Inveon Research Workplace visualisation software package (Siemens Preclinical Solution, Knoxville, TN).

### Statistical methods

Comparison of ^125^I-p5 and ^123^I-p5R or ^123^I-p5 and ^125^I-p5R biodistribution in mouse tissues was performed by using a paired, two-tailed Students t-test. Significance was set at *p*<0.05 and all analyses were conducted using SPSS Version 19 (Armonk, NY: IBM Corp).

## Results

**Figure 1:**
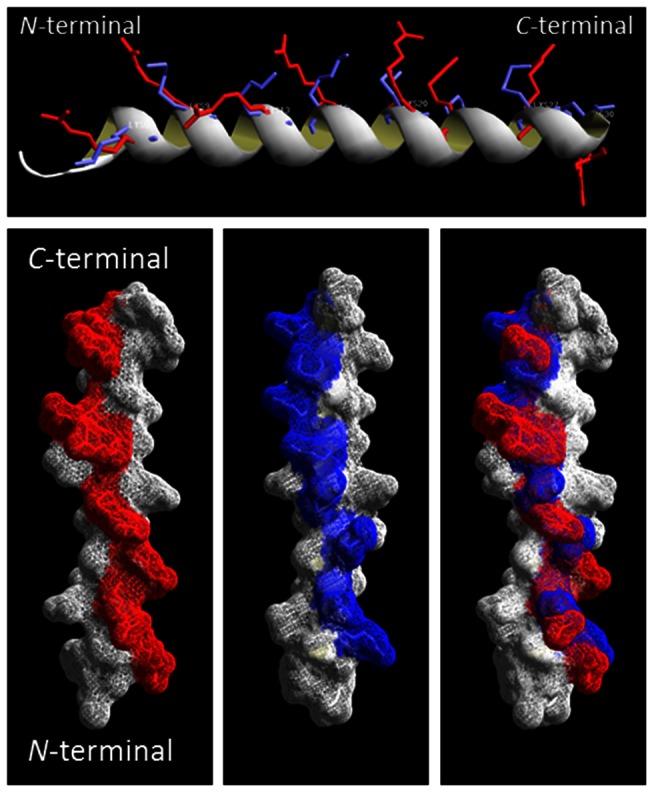
Peptides p5 and p5R predicted to adopt an α-helical secondary structure. Representations of peptides p5 with lysine side chains (blue) and p5R with arginine residues (red) overlayed showing α-helix backbone (upper) and as surface renderings. Models were predicted using iTASSER, and PDB files were rendered using DeepView/Swiss-PdbViewer v4.0.4 (www.expasy.org/spdbv/).

Peptides p5 and p5R each have 31 amino acids, a net charge of +8 and a pI of >10 (Table 1). The secondary structure was predicted based on the database-matching algorithms by using the iTASSER software [26], [27] to adopt an α-helical secondary structure (Fig. 1); however, the AGADIRms helix/coil transition algorithm [28], [29] predicted only 6.5% and 15.9% α-helix content for p5 and p5R, respectively (Table 1). Both peptides contain the heptad repeat (BxxBxxx) of amino acids with the basic residues (B), Lys and Arg, at positions *a* and *d* in p5 and p5R, respectively, which results in a nearly linear array of charge along a single face of the alpha helix (Figs. 1 A–C). When overlaid, the predicted positions of the basic amino acid side chains were similar for p5 and p5R (Fig.1 D).

**Table 1:** Primary structure and physical properties of peptide p5 and p5R.

	Peptide p5	Peptide p5R
Amino acid Sequence	GGGYS KAQKA QAKQA KQAQK AQKAQ AKQAK Q	GGGYS RAQRA QARQA RQAQR AQRAQ ARQAR Q
MW (Da)^a^	3257.7	3481.8
pI^a^	10.54	12.48
Net charge	+8	+8
Helix content^b^	6.47%	15.9%

a http://web.expasy.org/protparam/.

b pH 7, 0.1 ionic strength, 278 K: http://agadir.crg.es.

**Figure 2:**
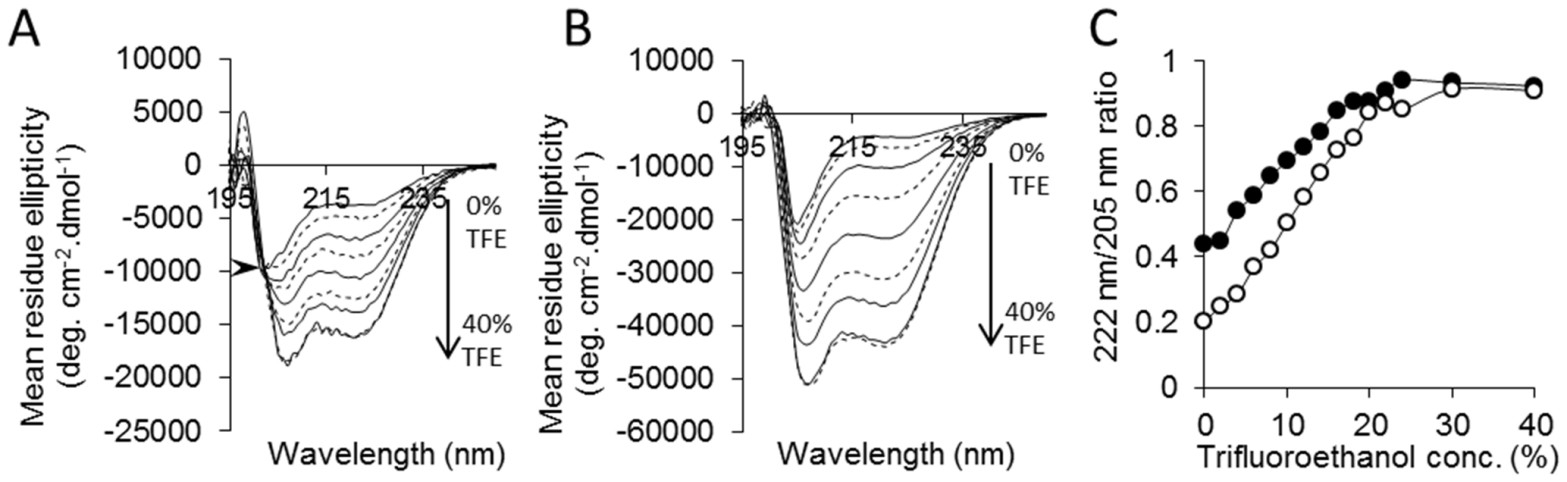
Peptide p5R exhibits enhanced α-helical secondary structure. CD spectra of peptides p5R (A – arrow shows isodichroic point) and p5 (B) in increasing concentrations of trifluoroethanol (TFE). The ratio of 222 nm and 205 nm was calculated (C) as a measure of α-helical content for p5 (○) and p5R (•). The 222 nm/205 nm ratio for human serum albumin in PBS was 1.0 (not shown).

The helicity of the peptides was assessed by using CD spectroscopy with varying concentrations of TFE (Figs. 2A & B). The CD spectra of both peptides contain minima at ∼205 nm and ∼220 nm but exhibit weak maxima at 195 nm indicative of a mix of α-helix and random coil. Helicity was assessed by calculating the ratio of the mean residue elipticity at 222 nm and 205 nm (Fig. 2C). Based on a CD spectrum of bovine serum albumin [30], a 222 nm/205 nm ratio of 1 was presumed to represent ∼100% helical structure. Both the p5 and p5R CD spectra ratios approached 1 as the concentration of TFE was increased to 40% by volume; however, in PBS alone, p5R exhibited 2-fold more helicity than p5 (Fig. 2C). As the TFE concentration was increased, the helicity of both peptides increased and was maximal and equivalent at 30% TFE (Fig. 2C). The spectra of peptide p5R (Fig. 2A) shows an isodichroic point (arrow) indicating a two state coil-to-helix transition. No such point was obvious in the p5 spectra.

**Figure 3:**
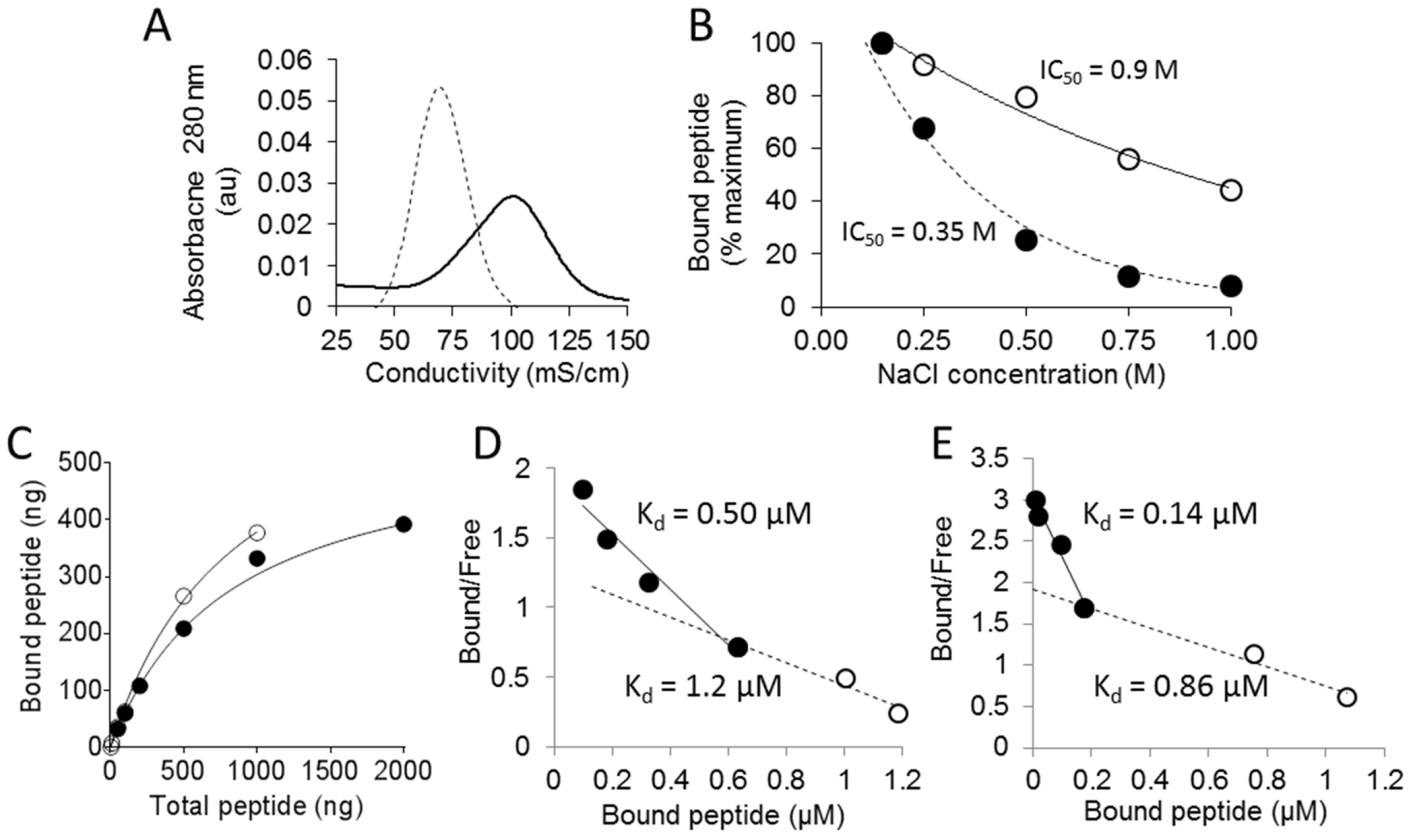
Peptide p5R binds immobilized heparin and AA amyloid tissue homogenate with high affinity. (A) Peptides p5R (solid) and p5 (dashed) eluted from a heparin-agarose column with peak conductivities of 100 mS/cm (∼1.3 M NaCl) and 70 mS/cm (∼0.9 M NaCl), respectively. (B) The concentration of NaCl required to inhibit 50% binding of peptide to AA amyloid-laden tissues (IC_50_) was 0.9 M and 0.35 M for p5R (gray) and p5 (black), respectively. (C) Binding of p5 (open) and p5R (closed) to AA amyloid-laden tissue homogenate assayed by addition of 5 ng of ^125^I-p5R with increasing concentrations of unlabeled homologous peptide. Scatchard plot of p5 (D) and p5R (E) binding to AA tissue homogenate. Data were fitted using 2 linear trendlines. K_d_ was determined as 1/K_a_ (slope  = –K_a_).

The relative affinity of the p5 and p5R peptides for heparin was assessed *in vitro* using a heparin-derivatized agarose column (Fig. 3A) Reactivity for murine AA amyloid was measured by the binding of radioiodinated peptides to hepatic AA amyloid in the presence of increasing NaCl concentrations (Fig. 3B). Even though the net charge of both peptides is 8+, peptide p5R bound with greater avidity to the heparin-agarose column as indicated by the greater amount of NaCl required to elute the peptide (i.e., peak values of 70 mS/cm [∼0.9 M NaCl] vs 100 mS/cm [∼1.3 M NaCl] for p5 and p5R, respectively – Fig. 3A). Similarly, the estimated IC_50_ of NaCl (the molar concentration required to inhibit 50% of the peptide binding) for the interaction of peptides with AA amyloid-laden liver tissue homogenate was ∼0.35 M and ∼0.9 M for p5 and p5R, respectively (Fig. 3B). Binding curves (Fig, 3C) and Scatchard plots were generated using 5 ng of radiolabeled p5 and p5R added to AA tissue homogenate in the presence of increasing concentrations of unlabeled peptide up to 2 µg or 1 µg for p5 and p5R, respectively (Figs. D and E). Scatchard analyses revealed 2 binding sites with AA liver homogenate for both peptides (Figs, 3D and 3E), with K_d_ values of 0.5 µM and 0.14 µM for the high affinity binding of p5 and p5R, respectively. The low affinity binding ∼ 1 µM for each peptide was essentially equivalent.

**Figure 4:**
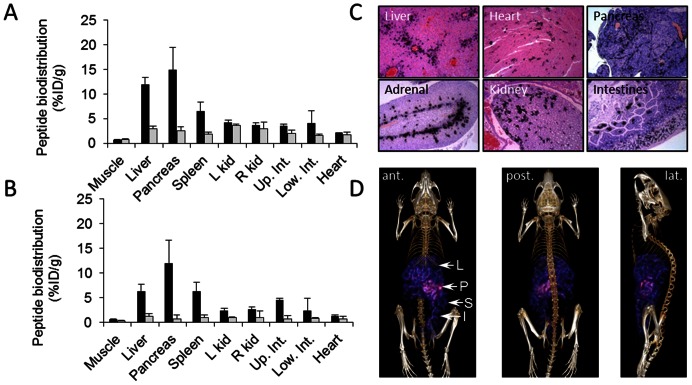
Peptide ^125^I-p5R specifically visualizes visceral AA amyloid *in vivo*. The biodistribution of radiolabeled peptide p5R in mouse tissues from healthy WT mice (grey) or AA amyloidotic mice (black) expressed as % injected dose per gram of tissue (%ID/g) at 1 h (A) and 4 h (B) post-injection (mean + SD, *n* = 3). (C) Specific localization of ^125^I-p5R peptide with amyloid was confirmed by micro-autoradiography where peptide was evidenced by the presence of black silver grains (original mag. 80x). (D) SPECT/CT imaging (anterior, posterior and lateral views) of mice at 2 h pi showed uptake of ^125^I-p5R in the liver (L), spleen (S), intestine (I), and pancreas (P).

For comparison with published data for p5 peptide, the amyloid reactivity of peptide p5R was assessed *in vivo* using the transgenic H2/IL-6 murine model of systemic visceral AA amyloidosis. In mice with moderate to severe AA amyloidosis (3 – 4+), the biodistribution of the peptide at 1 h and 4 h post injection (pi) was indicative of the distribution of amyloid in diseased mice (Figs. 4A & B). Notably, there was significant radioactivity seen in the liver, spleen, pancreas, and intestines as compared to the WT mice at both 1 h and 4 h pi. At both time points, the major uptake of radiolabeled p5R was the pancreas (>10% ID/g) followed by the liver and spleen (5 – 10% ID/g). The specificity of the peptide binding within the tissues and organs was assessed by using micro-autoradiography (Fig. 4C). The distribution of radiolabeled peptide, evidenced by the presence of black silver grains, co-registered with the presence of amyloid deposits seen in Congo red-stained consecutive tissue sections (not shown). The ^125^I-p5R peptide tracer bound AA amyloid in all tissues evaluated; notably, those deposits occupying the hepatic sinusoids, the myocardium, pancreatic microvasculature, zona reticularis of the adrenal gland, renal papilla, and intestinal villi (Fig. 4C). There was no evidence of ^125^I-p5R associated with healthy, amyloid-free tissues with the exception of the hyaline cartilage in the tracheal rings (presumably hypersulfated chondroitin sulfate), where mild uptake was observed (not shown).

Retention of the ^125^I-p5R in mice with AA was sufficient in the liver (L), pancreas (P), spleen (S) and intestines (I) to allow visualization by using SPECT/CT imaging (Fig. 4D). Notably, in the SPECT images, the pancreas exhibited the greatest concentration of activity, which correlated well with biodistribution findings (Figs. 4A & B). In contrast to previous SPECT imaging studies of AA amyloid in the H2/IL-6 mice using ^125^I-p5 [17], the spleen in these mice retained a remarkably low amount of radiolabeled p5R relative to the other organs, although the total amyloid burden measured by Congo red staining was similar.

**Figure 5:**
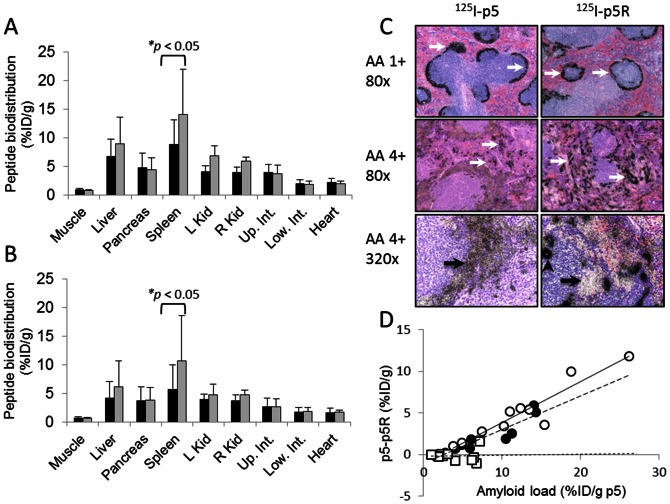
The distribution of peptide p5R differs from p5 in AA mice *in vivo*. Dual energy biodistribution studies of (A) ^125^I-p5R (black) and ^123^I-p5 (gray) and (B) ^123^I-p5R (black) and ^125^I-p5 (gray) in individual mice with moderate-to-severe AA amyloid (mean + SD, *n* = 5). (C) The microdistribution of ^125^I-p5 and ^125^I-p5R in separate mice with weak (1+) and strong (4+) AA assessed by autoradiography. Arrows indicate the presence of “large” AA splenic amyloid. (D) The difference between ^125^I-p5 and ^125^I-p5R uptake (i.e. %ID/g p5 – %ID/g p5R) in the spleen (○), liver (•) and pancreas (□) relative to amyloid load (%ID/g ^125^I-p5).

Faced with the counter-intuitive observation that peptide p5R had a greater affinity for heparin (Fig. 3A) and *ex vivo* AA amyloid (Fig. 3B), yet the reactivity with splenic AA amyloid *in vivo* appeared markedly reduced relative to studies with peptide p5, we examined the binding of both peptides in the same subject by using dual-energy biodistribution analyses (Fig. 5). Individual H2/IL-6 mice with moderate to severe AA amyloid (3 – 4+) received ^125^I-p5R and ^123^I-p5 or ^125^I-p5 and ^123^I-p5R. Biodistribution measurements at 2 h pi (Fig. 5A & B) showed significantly greater retention of p5 in splenic AA amyloid as compared to peptide p5R (*p* = 0.045, Fig. 5A; p = 0.032, Fig. 5B). There was no significant difference (*p* > 0.05) in the retention of peptides p5 and p5R in hepatic or pancreatic AA amyloid deposits.

In an attempt to discern the reason for this discrepancy, we analyzed micro-autoradiographs of spleens from mice with 1+ or 4+ AA amyloid that had been injected with ^125^I-p5 or ^125^I-p5R. In mice with mild (1+) amyloid, the micro-distribution was uniform throughout the perifollicular AA amyloid deposits for both peptides (Fig. 5C upper, arrows); however, in mice with 4+ splenic AA amyloid, the peptide uptake pattern varied markedly between p5 and p5R when viewed at 80x (Fig. 5C panel). The ^125^I-p5 remained fairly uniformly distributed throughout the amyloid with scant areas of punctate uptake (arrows). In contrast, the ^125^I-p5R peptide appeared to be focally deposited (arrows) with areas of apparently dense, relatively avascular amyloid that were devoid of significant p5R uptake. This phenomenon was more readily seen in images taken using a magnification of 320x (Fig 5C. bottom). The ^125^I-p5 peptide was uniformly distributed in the perfollicular amyloid (arrow); however, in contrast, there were regions of AA that lacked ^125^I-p5R (arrow) and numerous areas of dense focal uptake (arrowhead) that appeared to correlate with the deposition of perivascular or perisinusoidal AA amyloid.

To further parameterize this phenomenon, we examined the difference in retention of radioiodinated p5 and p5R (%ID/g) vs the amount of amyloid in the organ – estimated by the accumulation of p5 peptide (%ID/g; Fig. 5D). As the amount of splenic or hepatic AA amyloid increased the more p5 peptide was bound relative to p5R in that organ. For example, in a mouse with a splenic amyloid load equivalent to 20% ID/g of p5 peptide, the p5 peptide was retained 10% ID/g more than peptide p5R. This phenomenon was not observed for the pancreas, i.e., regardless of the pancreatic amyloid load, the amount of p5 or p5R retained in that tissue was nearly equivalent.

**Figure 6:**
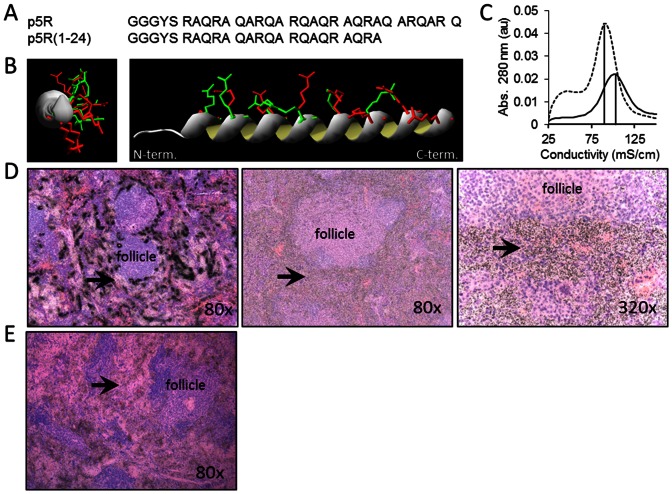
Peptide ^125^I-p5R(1-24) microdistribution is uniform relative to ^125^I-p5R in splenic AA amyloid. Primary (A) and predicted secondary structure (B) comparison of peptides p5R (red arg side chains) and p5R(1-24) (green arg side chains). (C) Peptide p5R(1-24) eluted from a heparin column with conductivity of 90 mS./cm at the maximum. (D) Microautoradiographs of ^125I^I-p5R (left) and ^125^I-p5R(1-24) (center and right) in 4+ AA amyloid-laden spleen tissue sections.. (E) Microautoradiograph of splenic distribution of ^125^I-p5R (∼ 10 µg) when mixed with 1 mg of unlabeled p5R prior to iv injection. Original magnification as shown. Arrows indicate sites of peptide-free amyloid in (left) and diffuse (non-punctate) uptake of peptide in amyloid (center and right).

The difference in microdistribution pattern could be due to differences in binding of the arginine and lysine side chains with the target in the amyloid; however, it also could be explained by the higher affinity and binding of p5R to the first amyloid target encountered, thereby depleting the probe from penetrating deeper into the dense amyloid deposit. To test this latter hypothesis, we first constructed peptide p5R(1-24), a 24-mer variant lacking the N-terminal 7 amino acids and with a reduced net charge of +6 (Fig. 6A). This peptide has the same predicted positioning of arginine side chains along a single face of the helix (Fig. 6B), but it exhibited a lower affinity for immobilized heparin relative to p5R due to the loss of 2 arginine residues (∼ 90 mS/cm Fig. 6C). In contrast to the dense, focal splenic micro-distribution of ^125^I-p5R peptide (Fig. 6D left) in mice with moderate AA amyloidosis, the ^125^I-p5R(1-24) peptide was markedly diffuse (Figs. 6D center and right). No dense, focal perifollicular retention of ^125^I-p5R(1-24) was seen in the spleens of mice with AA. The micro-distribution of ^125^I-p5R(1-24) in the spleen of mice with moderate AA was similar to that of the ^125^I-p5 peptide (Fig. 5C). In a second approach, the ^125^I-p5R was mixed with 1 mg of unlabeled p5R peptide before iv tail vein injection. The distribution of ^125^I-p5R, as shown by micro-autoradiography, was markedly more diffuse and extended into the dense perifollicular splenic AA amyloid (Fig. 6E), which was in stark contrast to the ^125^I-p5R injected alone (Fig. 6D, left).

## Discussion

The detection of amyloid by using non-invasive molecular imaging techniques has experienced a resurgence in the last 5 years with the realization that the ability to quantitatively and longitudinally assess amyloid load in the brains of patients with mild cognitive impairment (MCI) provides valuable prognostic information [31]. Similarly, the ability to detect cerebral amyloid in patients with mild to moderate Alzheimer’s disease will invariably assist with making differential diagnoses, stratification of patients for novel therapies and for monitoring response to therapy, both in the clinic and in amyloid-focused clinical trials. Recently, Amyvid™ (also known as, Av45 or florbetapir) was approved by the US Food and Drug Administration (FDA) and in the European Union (EU) as an ^18^F-labeled PET radiotracer for the specific detection of amyloid in the brains of patients with MCI and early AD [32]. None of the Aβ amyloid-reactive probes have general application for imaging peripheral amyloid.

In other amyloid-related diseases, only ^123^I-SAP has been used routinely within the clinic to detect the presence of amyloid in visceral organs principally in patients with AL and AA amyloidosis, but patients with other rarer forms of spontaneous and hereditary amyloid-related conditions have been studied [14], [33], [34]. SAP imaging has not been approved by the US FDA as an imaging technique and is only routinely performed in 3 centers in the EU. Even though SAP scans provide useful information, there is no evidence that ^123^I-SAP scintigraphy or SPECT imaging is sufficiently sensitive to visualize the often scant and diffuse, but debilitating amyloid within the heart. In addition, pancreatic amyloid composed of islet-associated polypeptide (IAPP) that is common in patients with type 2 diabetes and may have etiologic significance, has yet to be imaged using SAP or any other radiotracer.

Given the potential importance of amyloid imaging for the purpose of prognostication, patient stratification and monitoring response the therapy, we evaluated a panel of heparin-reactive peptides for their ability to specifically bind the hypersulfated heparan sulfate proteoglycans that are a ubiquitous component of all cerebral and visceral amyloid deposits and, thereby, provide a means for imaging the deposits *in vivo*. A screen of 7 basic peptides with net positive charges ranging +6 to +16 yielded peptide p5 (+8; [35]) as the optimal imaging agent based on the uptake of ^125^I-labeled peptide in amyloid laden organs (% injected dose per gram of tissue) and the microdistribution of the ^125^I-p5 peptide in the amyloid as evidenced in microautoradiographs [17]. The efficacy of peptide p5, relative to the other reagents, was posited to be due to the ability of this peptide to adopt a polar α-helical configuration that aligned all the charged lysine side chains along one face of the helix [36]. This distribution of charge has been shown to be optimal for the reactivity of this peptide with soluble heparin [36], [37] due to the availability of 8 basic side chains that can interact electrostatically with the linear charges on heparin and hypersulfated heparan sulfate. The potential for multivalent interaction greatly enhances the affinity of the binding and specificity is bestowed by the spatial arrangements of the positive charges on one face of the α helix coinciding with the positions of the corresponding negative charges on the target. Although p5 and p5R have similar predicted positive charge arrangements when in α helical form (Fig.1), substitution of the lysine residues in p5 by arginine to produce p5R resulted in a much greater affinity of p5R for heparin, likely due to its inherently greater propensity for helicity in PBS (Fig. 2). Similar data has been presented for related peptides where the lysine variant bound soluble heparin with a *K*_d_ of 640 nM as compared to 3.4 nM for the arginine-substituted peptide [36].

The arginine-substituted form of p5 (p5R) bound with greater affinity, as compared to p5, to immobilized heparin as well as murine AA amyloid-laden liver homogenate, suggesting that the same linear electrostatic interactions were important for both heparin and amyloid binding (presumably via the fibril-associated heparan sulfate moieties). Both peptides exhibit modest α-helical structure in physiological saline, with p5R having 2-fold more helix as evidenced in CD spectra. In the presence of trifluoroethanol (TFE), which promotes the formation of helical secondary structure [38], both peptides became more structured reaching the same plateau value of 222 nm/205 nm ratio at 30– 40% TFE. A peptide similar in sequence to peptide p5 adopts an α-helical conformation in the presence of soluble heparin [35], [36], indicating that the presence of natural binding partners can promote and stabilize a helical, ligand-binding conformation.

**Figure 7:**
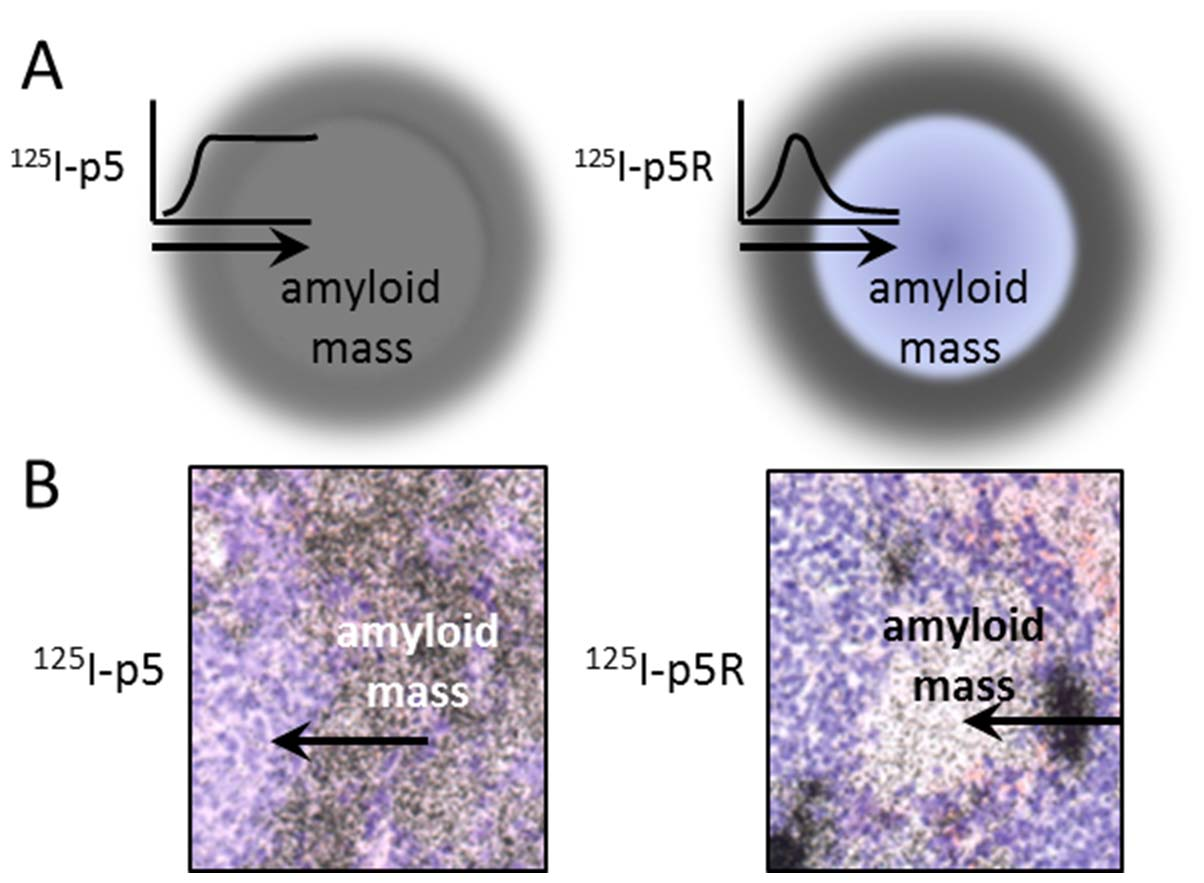
The binding-site barrier effect. (A) Schematic representation of the diffuse accumulation of p5 throughout the amyloid lesion. In contrast, peptide p5R accumulates as non-uniform (focal) deposits at the periphery of the amyloid mass. (B) Microautoradiographs of ^125^I-p5 and ^125^I-p5R that exemplify the consequence of a binding-site barrier on the amyloid-uptake of radiolabeled peptides.

Given the enhanced helicity and resulting increased affinity for amyloid, it was anticipated that the radiolabeled peptide ^125^I-p5R would provide enhanced amyloid imaging *in vivo.* Based on biodistribution studies and SPECT imaging, the p5R peptide bound AA amyloid in mice and generated images indiscernible from images that had been produced using ^125^I-p5 peptide [17], [18]. However, analysis of the microdistribution of ^125^I-p5R peptide in mice with AA amyloid revealed that, even though the “small” discrete amyloid lesions that are common e.g., in the liver, heart, pancreas and adrenal were evenly covered by radiolabeled peptide, the large expanses of avascular, dense amyloid seen in the spleens of highly diseased animals were variably stained. This phenomenon was most evident in mice with extensive (3+ – 4+; [19]) splenic amyloid but, interestingly, not in mice with (1+ – 2+) amyloid in the spleen which suggests that this effect is not due to the lack of a binding target in the splenic amyloid. It is likely that the enhanced affinity of the p5R peptide for amyloid resulted in a binding-site barrier effect which causes a non-uniform distribution of reagent within the target site and was first observed for tumor-targeting monoclonal antibodies [39]–[42]. In these cases, the antibodies, as with the p5R peptide, are delivered to the target via the vasculature, and as the reagent passes through the vascular wall, it binds with high affinity to the first target encountered and does not penetrate into the center of the lesion [41], [42]. The affinity of p5R for AA amyloid-laden tissue homogenate *in vitro* (K_d_ estimated to be 0.15 µM) is only ∼4-fold greater than that of p5 (K_d_ ∼ 0.5 µM); however even this modest increase can lead to differences of more than 40 µm of tumor penetration from the blood vessel when mAbs are studied [41]. This process results in a dense accumulation of peptide at the regions of amyloid that are in the immediate vicinity of the supplying vasculature with a decreasing density of peptide in the “center” of the amyloid lesion. For probes of lower affinity, some probe can bypass the primary binding site, penetrating farther into dense target before finding a partner. This is shown schematically and with 2 examples in Fig. 7. One consequence of this effect is that larger amyloid masses or those with low surface area to volume ratios will likely accumulate less p5R peptide as compared to p5. Indeed, we demonstrated a significantly lower amount of ^125^I-p5R in the spleens of mice with >3+ amyloid as compared to peptide ^125^I-p5 (*p* = 0.032). The binding-site barrier effect for antibodies was overcome by using F(ab) fragments which are capable of only monovalent interactions and, therefore, have a lower affinity for the target as compared to bivalent Abs [43]. To mimic this, we first synthesized a C-terminally truncated p5R variant, p5R(1-24), that lacked 2 arginine residues. This shorter electronic signature (6 charges vs 8 charges) resulted in a relatively lower affinity for heparin. The microdistribution of this peptide in the expansive splenic AA amyloid deposits was similar to the diffuse accumulation seen with ^125^I-p5, supporting the hypothesis that the enhanced affinity of p5R for amyloid resulted in a barrier effect. This effect hindered penetration, altered the distribution in the amyloid and significantly decreased the accumulation of peptide in large amyloid deposits relative to that of p5. Enhanced penetration of p5R into the expansive splenic amyoid was also achieved by increasing the peptide concentration to 1 mg, which resulted in a saturation of the perivascular amyloid allowing p5R to penetrate more uniformly into the amyloid mass.

Although these observations have been made in the murine model of AA amyloidosis, this phenomenon suggests that translation of the p5R peptide for clinical detection of amyloid in patients should be considered with caution as it may not provide quantitative images of amyloid load in patients with excessive disease. These patients present with very high amyloid burdens of liver and spleen, and amyloid load is readily detected by imaging. Many proposed therapies for peripheral amyloid focus on reducing amyloid burden. If the goal of non-invasive imaging is monitoring response to therapy, a one to one correlation of probe binding to amyloid load is essential; however, in these therapies applied to highly diseased patients, one would expect changes in the surface area to volume ratio to accompany disease progression or regression. Thus, for quantitative imaging, a lower affinity probe should give more linear results. In cases where the amyloid under investigation is discrete and in close proximity to the vasculature, for example in patients with Aβ-associated cerebral amyloid angiopathy (associated with an increased risk of stroke; [44]) or with the pancreatic islets of patients with type 2 diabetes, then imaging with a high affinity probe such as peptide p5R may be more appropriate. Our results demonstrate the excellent specificity of either p5 or p5R for AA amyloid deposits in the huIL-6 mice relative to WT mice. Our prediction that p5R would have a higher affinity for heparin and for AA amyloid held true; however, this higher affinity compounded correlation of SPECT images with amyloid load. We conclude that p5 is an optimal imaging agent for non-invasive detection and quantitation of some peripheral amyloids (such as AA and AL). For less dense and more vascular forms (Aβ, IAPP etc.), the higher affinity p5R should be superior. These ideas need to be tested in appropriate patient populations.

## References

[1] Blancas-Mejia LM, Ramirez-Alvarado M (2013) Systemic Amyloidoses. Annu Rev Biochem. 10.1146/annurev-biochem-072611-130030 PMC4044913 23451869

[2] Guan J, Mishra S, Falk RH, Liao R (2012) Current perspectives on cardiac amyloidosis. Am J Physiol Heart Circ Physiol 302: H544–552. 22058156 10.1152/ajpheart.00815.2011 PMC3353775

[3] Pepys MB (2006) Amyloidosis. Annu Rev Med 57: 223–241. 16409147 10.1146/annurev.med.57.121304.131243

[4] Skaper SD (2012) Alzheimer's disease and amyloid: culprit or coincidence? Int Rev Neurobiol 102: 277–316. 22748834 10.1016/B978-0-12-386986-9.00011-9

[5] Antoni G, Lubberink M, Estrada S, Axelsson J, Carlson K, et al (2013) In Vivo Visualization of Amyloid Deposits in the Heart with 11C-PIB and PET. J Nucl Med 54: 213–220. 23238792 10.2967/jnumed.111.102053

[6] Glaudemans AW, Slart RH, Zeebregts CJ, Veltman NC, Tio RA, et al (2009) Nuclear imaging in cardiac amyloidosis. Eur J Nucl Med Mol Imaging 36: 702–714. 19156411 10.1007/s00259-008-1037-1

[7] Schaadt BK, Hendel HW, Gimsing P, Jonsson V, Pedersen H, et al (2003) 99mTc-aprotinin scintigraphy in amyloidosis. J Nucl Med 44: 177–183. 12571206

[8] Smyth DR, Tsopelas C (2005) An improved (99m)Tc-aprotinin kit formulation: quality control analysis of radiotracer stability and cold kit shelf life. Nucl Med Biol 32: 885–889. 16253814 10.1016/j.nucmedbio.2005.06.006

[9] Puille M, Altland K, Linke RP, Steen-Muller MK, Kiett R, et al (2002) 99mTc-DPD scintigraphy in transthyretin-related familial amyloidotic polyneuropathy. Eur J Nucl Med Mol Imaging 29: 376–379. 12002714 10.1007/s00259-001-0730-0

[10] Rapezzi C, Guidalotti P, Salvi F, Riva L, Perugini E (2008) Usefulness of 99mTc-DPD scintigraphy in cardiac amyloidosis. J Am Coll Cardiol 51: : 1509–1510; author reply 1510. 10.1016/j.jacc.2007.12.038 18402909

[11] Gejyo F, Kazama JJ, Hasegawa S, Nishi S, Arakawa M, et al (1995) 131I-beta 2-microglobulin scintigraphy in patients with dialysis amyloidosis. Clin Nephrol 44 Suppl 1S14–18. 8608655

[12] Ketteler M, Koch KM, Floege J (2001) Imaging techniques in the diagnosis of dialysis-related amyloidosis. Semin Dial 14: 90–93. 11264773 10.1046/j.1525-139x.2001.00025.x

[13] Hachulla E, Grateau G (2002) Diagnostic tools for amyloidosis. Joint Bone Spine 69: 538–545. 12537260 10.1016/s1297-319x(02)00449-9

[14] Hawkins PN, Pepys MB (1995) Imaging amyloidosis with radiolabelled SAP. Eur J Nucl Med 22: 595–599. 7498219 10.1007/BF01254559

[15] Hazenberg BP, van Rijswijk MH, Piers DA, Lub-de Hooge MN, Vellenga E, et al. (2006) Diagnostic performance of 123I-labeled serum amyloid P component scintigraphy in patients with amyloidosis. Am J Med 119: 355 : e315–324. 10.1016/j.amjmed.2005.08.043 16564782

[16] Westermark P (2011) Amyloid in the islets of Langerhans: thoughts and some historical aspects. Ups J Med Sci 116: 81–89. 21486192 10.3109/03009734.2011.573884 PMC3078536

[17] Wall JS, Richey T, Stuckey A, Donnell R, Macy S, et al (2011) In vivo molecular imaging of peripheral amyloidosis using heparin-binding peptides. Proc Natl Acad Sci U S A 108: E586–594. 21807994 10.1073/pnas.1103247108 PMC3161606

[18] Wall JS, Richey T, Williams A, Stuckey A, Osborne D, et al (2012) Comparative analysis of peptide p5 and serum amyloid P component for imaging AA amyloid in mice using dual-isotope SPECT. Mol Imaging Biol 14: 402–407. 22042488 10.1007/s11307-011-0524-0 PMC3664664

[19] Wall JS, Richey T, Macy S, Heidel E, Wooliver C, et al (2013) A novel method for quantifying peripheral tissue amyloid load by using the radiolabeled amyloidophilic peptide, p5. Amyloid 20: 21–26. 23327342 10.3109/13506129.2012.757216 PMC3710708

[20] Solomon A, Weiss DT, Schell M, Hrncic R, Murphy CL, et al (1999) Transgenic mouse model of AA amyloidosis. Am J Pathol 154: 1267–1272. 10233864 10.1016/S0002-9440(10)65378-3 PMC1866549

[21] Wall JS, Richey T, Allen A, Donnell R, Kennel SJ, et al (2008) Quantitative tomography of early-onset spontaneous AA amyloidosis in interleukin 6 transgenic mice. Comp Med 58: 542–550. 19149411 PMC2710755

[22] Axelrad MA, Kisilevsky R, Willmer J, Chen SJ, Skinner M (1982) Further characterization of amyloid-enhancing factor. Lab Invest 47: 139–146. 7109539

[23] Wall JS, Paulus MJ, GLeason S, Gregor J, Solomon A, et al (2006) Micro-Imaging of Amyloid in Mice. Methods in Enzymology 412: 161–182. 17046658 10.1016/S0076-6879(06)12011-X PMC1805492

[24] Magota K, Kubo N, Kuge Y, Nishijima K, Zhao S, et al (2011) Performance characterization of the Inveon preclinical small-animal PET/SPECT/CT system for multimodality imaging. Eur J Nucl Med Mol Imaging 38: 742–752. 21153410 10.1007/s00259-010-1683-y

[25] Feldkamp LA, Davis LC, Kress JW (1984) Practical Cone-Beam Algorithm. Journal of Opt Soc Am 1: 612–619.

[26] Roy A, Kucukural A, Zhang Y (2010) I-TASSER: a unified platform for automated protein structure and function prediction. Nat Protoc 5: 725–738. 20360767 10.1038/nprot.2010.5 PMC2849174

[27] Zhang Y (2008) I-TASSER server for protein 3D structure prediction. BMC Bioinformatics 9: 40. 18215316 10.1186/1471-2105-9-40 PMC2245901

[28] Munoz V, Serrano L (1994) Elucidating the folding problem of helical peptides using empirical parameters. Nat Struct Biol 1: 399–409. 7664054 10.1038/nsb0694-399

[29] Munoz V, Serrano L (1997) Development of the multiple sequence approximation within the AGADIR model of alpha-helix formation: comparison with Zimm-Bragg and Lifson-Roig formalisms. Biopolymers 41: 495–509. 9095674 10.1002/(SICI)1097-0282(19970415)41:5<495::AID-BIP2>3.0.CO;2-H

[30] Sjoholm I, Grahnen A (1972) Circular dichroism studies on the binding of L-tryptophan to human serum albumin. FEBS Lett 22: 109–112. 11946574 10.1016/0014-5793(72)80232-1

[31] Johnson KA, Sperling RA, Gidicsin CM, Carmasin JS, Maye JE, et al.. (2013) Florbetapir (F18-AV-45) PET to assess amyloid burden in Alzheimer's disease dementia, mild cognitive impairment, and normal aging. Alzheimers Dement. 10.1016/j.jalz.2012.10.007 PMC3800236 23375563

[32] Yang L, Rieves D, Ganley C (2012) Brain amyloid imaging--FDA approval of florbetapir F18 injection. N Engl J Med 367: 885–887. 22931256 10.1056/NEJMp1208061

[33] Hawkins PN (2002) Serum amyloid P component scintigraphy for diagnosis and monitoring amyloidosis. Curr Opin Nephrol Hypertens 11: 649–655. 12394612 10.1097/00041552-200211000-00013

[34] Pepys MB (1992) Amyloid P component and the diagnosis of amyloidosis. J Intern Med 232: 519–521. 1474357 10.1111/j.1365-2796.1992.tb00630.x

[35] Jayaraman G, Wu CW, Liu YJ, Chien KY, Fang JC, et al (2000) Binding of a de novo designed peptide to specific glycosaminoglycans. FEBS Lett 482: 154–158. 11018540 10.1016/s0014-5793(00)01964-5

[36] Rullo A, Nitz M (2010) Importance of the spatial display of charged residues in heparin-peptide interactions. Biopolymers 93: 290–298. 19885920 10.1002/bip.21339

[37] Nitz M, Rullo A, Ding MX (2008) Heparin dependent coiled-coil formation. Chembiochem 9: 1545–1548. 18506865 10.1002/cbic.200800056

[38] Crandall YM, Bruch MD (2008) Characterization of the structure and dynamics of mastoparan-X during folding in aqueous TFE by CD and NMR spectroscopy. Biopolymers 89: 197–209. 18008325 10.1002/bip.20891

[39] Fujimori K, Covell DG, Fletcher JE, Weinstein JN (1990) A modeling analysis of monoclonal antibody percolation through tumors: a binding-site barrier. J Nucl Med 31: 1191–1198. 2362198

[40] Juweid M, Neumann R, Paik C, Perez-Bacete MJ, Sato J, et al (1992) Micropharmacology of monoclonal antibodies in solid tumors: direct experimental evidence for a binding site barrier. Cancer Res 52: 5144–5153. 1327501

[41] Rudnick SI, Lou J, Shaller CC, Tang Y, Klein-Szanto AJ, et al (2011) Influence of affinity and antigen internalization on the uptake and penetration of Anti-HER2 antibodies in solid tumors. Cancer Res 71: 2250–2259. 21406401 10.1158/0008-5472.CAN-10-2277 PMC3077882

[42] van Osdol W, Fujimori K, Weinstein JN (1991) An analysis of monoclonal antibody distribution in microscopic tumor nodules: consequences of a "binding site barrier". Cancer Res 51: 4776–4784. 1893370

[43] Fujimori K, Covell DG, Fletcher JE, Weinstein JN (1989) Modeling analysis of the global and microscopic distribution of immunoglobulin G, F(ab')2, and Fab in tumors. Cancer Res 49: 5656–5663. 2790783

[44] Loitfelder M, Seiler S, Schwingenschuh P, Schmidt R (2012) Cerebral microbleeds: a review. Panminerva Med 54: 149–160. 22801432

